# Impact of mining projects on water and sanitation infrastructures and associated child health outcomes: a multi-country analysis of Demographic and Health Surveys (DHS) in sub-Saharan Africa

**DOI:** 10.1186/s12992-021-00723-2

**Published:** 2021-06-30

**Authors:** Dominik Dietler, Andrea Farnham, Georg Loss, Günther Fink, Mirko S. Winkler

**Affiliations:** 1 Department of Epidemiology and Public Health, Swiss Tropical and Public Health Institute, P.O. Box CH-4002, Basel, Switzerland; 2 University of Basel, P.O. Box CH-4001, Basel, Switzerland

**Keywords:** Child health, Diarrhea, Drinking water, Malnutrition, Natural resource extraction, Sanitation

## Abstract

**Background:**

Access to improved water and sanitation infrastructures are key determinants of health. The sub-Saharan African region in particular is lagging behind the ambitious goal of the 2030 Agenda for Sustainable Development to ensure universal access to improved and reliable water and sanitation for all (Sustainable Development Goal (SDG) 6). Large mining projects can promote economic growth and hence investments in water and sanitation infrastructures, but at the same time lead to rapid population growth and environmental degradation. In turn, these changes can pose risks and opportunities for child health (SDG 3). In this study we aim to quantify the impacts of mining projects on access to water and sanitation infrastructure as well as diarrhea and malnutrition among children using data from 131 Demographic and Health Surveys from sub-Saharan Africa.

**Results:**

From a sample of around 1.2 million households, data within the proximity of 52 mine-panels were selected for longitudinal analyses, resulting in 41,896 households and 32,112 children. Improvements in access to modern water and sanitation infrastructures after mine opening were much larger in households near mining sites than in comparison areas located further away (adjusted relative risk ratio (aRRR) water: 18.60, 95 % confidence interval (CI): 13.08–26.46 and aRRR sanitation: 2.56, 95 % CI: 1.32–4.99). However, these associations were weaker among poorer households. In areas close to the mining sites, stunting and underweight prevalence decreased more strongly upon mine opening (adjusted odds ratio (aOR) stunting: 0.62, 95 % CI: 0.43–0.90; aOR underweight: 0.55, 95 % CI: 0.36–0.84). No differential changes were seen for wasting and diarrhea. Large impact heterogeneity was observed both within and across countries.

**Conclusions:**

Our results suggest that the opening of mines is associated with improvements in access to modern water and sanitation infrastructures (SDG 6) as well as in some health outcomes (SDG 3). However, the large impact heterogeneity suggests that the assessment and management of mining-related impacts on communities should have an increased equity-focus, in order to “leave no one behind” in the work towards the 2030 Agenda for Sustainable Development. Overall, the findings of this study underscore that the resource extraction sector has the potential to make positive and substantial contributions towards achieving the SDGs.

**Supplementary Information:**

The online version contains supplementary material available at 10.1186/s12992-021-00723-2.

## Introduction

Despite major improvements in the provision of clean drinking water and improved sanitation infrastructures in the last decades, substantial gaps in access persist [1]. In 2017, 2.2 billion people lacked access to safely managed drinking water and 4.2 billion people lacked access to safely managed sanitation facilities [1]. The 2030 Agenda for Sustainable Development ambitiously demands “universal access to improved and reliable water and sanitation for all” (Sustainable Development Goal (SDG) 6) by 2030 [2]. Such improvements do however require substantial resources, which remain particularly scarce in world regions that are currently far from achieving SDG 6 [3].

Some of the poorest countries, particularly in sub-Saharan Africa (SSA), are extremely rich in mineral and metal resources, such as diamonds, gold, iron and copper [4]. The development of large mining projects creates unique opportunities for economic development, which in turn can promote better public and private infrastructures [5–9]. Investments at the community and household-level could for example include the expansion of drinking water distribution networks, protection of wells, septic systems or improved toilet facilities [5]. On the other hand, depending on the type of resources extracted, the mining technology applied and environmental management in place, extracting and processing minerals is highly water-intense and can lead to environmental pollution [10–14]. At the same time, mining projects can result in rapid population growth [15, 16]. Hence, mining projects can put additional strains on often already overburdened water and sanitation systems in affected communities [11, 12, 17, 18].

Evidence on the impacts of mining projects on water and sanitation infrastructures is inconclusive. A case study conducted in a mining area in Peru showed positive impacts on water and sanitation infrastructures, while other studies found negative impacts in Mali and Tanzania [19, 20]. In a study focusing on Ghana, Mali and Tanzania, no significant impact of mining activities on access to improved water and sanitation infrastructures was found [21].

Changes in water and sanitation infrastructures can potentially improve health and well-being, even with additional environmental pollution (SDG 3) [22, 23]. Children are particularly vulnerable to the health consequences of the lack of access to these infrastructures [24]. In low- and middle-income countries, a third of the childhood diarrhea burden is attributable to inadequate drinking water and one in five diarrhea cases are attributed to the lack of sanitation [23]. Repeated diarrheal episodes negatively impact the nutritional status of children, which increases their vulnerability to diarrheal infections. Improvements in water and sanitation infrastructure can help to break this “vicious cycle” by lowering the risk of diarrheal diseases and improving nutritional status in children[22, 25, 26].

The effect of mines on childhood diarrhea and malnutrition is not well understood. In a large sample of children around mines and ore smelters in multiple developing countries, von der Goltz et al. found that children in mining areas are taller for their age than children in comparable areas without mining projects [8]. Evidence from single-country analyses point at positive impacts of mining projects on child nutrition in Mali but negative effects in Tanzania [20], while in a case study in Zambia no effect was found [27]. Similarly, the findings on the impact on diarrheal diseases are inconclusive [20, 21, 28]. In Zambia, the burden of diarrhea-causing parasitic infections decreased in mining areas [28]. Other studies found diarrheal incidence to be lower in mining areas in Mali but unchanged in Tanzania or Ghana [20, 21].

In this study [29], we use the largest currently available dataset to systematically assess the impacts of large mining projects on access to water and sanitation infrastructure and associated health outcomes in sub-Saharan Africa. More specifically, we use a quasi-experimental difference-in-differences design to test whether mining projects affect access to water and sanitation infrastructures as well as whether these changes have an impact on water and sanitation-related child health outcomes.

## Methods

### Data

#### Demographic and Health Survey (DHS) data

The Demographic and Health Surveys (DHS) program has been conducting nationally-representative cross-sectional household surveys in low- and middle-income countries since the 1980 s [30]. Households are selected through a two-stage cluster sampling methodology. At the first stage, clusters (typically villages in rural areas or blocks in urban areas) are sampled using a probability proportional to population size strategy. At the second stage, all households are listed in the selected area and then 25–30 households are randomly selected for the interviews. This strategy allows to obtain a representative sample of households at the regional-level as well as for urban and rural areas. For most surveys, Global Positioning System (GPS) data of the clusters are available. To ensure the privacy of respondents, these coordinates are shifted at random up to 2 km for urban clusters and up to 5 km for rural clusters (10 km for 1 % of rural clusters). In the present study, all household and child data from surveys in sub-Saharan Africa with GPS data available as of March 2020 were included.

#### Mining

Data on the type and location of mines were derived from the Standard & Poor’s (S&P) Global Market Intelligence Mining Database [31]. The database contains the location and basic characteristics of all major mines in the world. Information on historic mining activities is provided in two ways. Firstly, the opening and closing years of the mines are reported. Secondly, annual extraction and production information since 1980 was available. The first operational year was set as the earlier of the reported opening year or the first year with reported production/extraction. Thus, the opening year marked the start of the operation phase of a mine, not including the construction phase. The last operation year was set as the last year with reported production/extraction unless a later closing year was explicitly reported. If both were not available and the mine was listed as “active” the last operation year was set as 2019. During the period between the first and last operation year the mines were considered operational. Furthermore, for longitudinal analysis, a variable was created indicating whether the mine was geographically isolated or located in proximity to other mines. Mines were considered as being isolated if they were at least 100 km away from other mines.

#### Data merging

To merge the mining with the DHS data, the locations of all DHS clusters and mines were mapped using ArcGIS Pro (Version 2.2.4, Environmental Systems Research Institute, Redlands, CA, USA). Information on the distance to the closest mine and their activity status between 1980 and 2019 were extracted at each cluster location.

Given that in urban areas, a broad variety of factors influence water and sanitation infrastructure access and child health, data from DHS clusters located in cities with more than 100,000 inhabitants were excluded. For this, the coordinates of the center points of cities in sub-Saharan Africa from Natural Earth [32] were integrated in the map. Around each city center, circles of different radii were drawn to represent their approximate boundaries. The size of the radii varied depending on the number of inhabitants (5 km for cities with 0.1–0.5 million inhabitants, 7.5 km for 0.5-1 million, 15 km for 1–5 millions and 40 km for more than 5 millions). These distances were determined by measuring the size of built-up area using satellite imagery over a random selection of cities in each category. DHS clusters within the city boundaries were excluded from analysis.

**Fig. 1:**
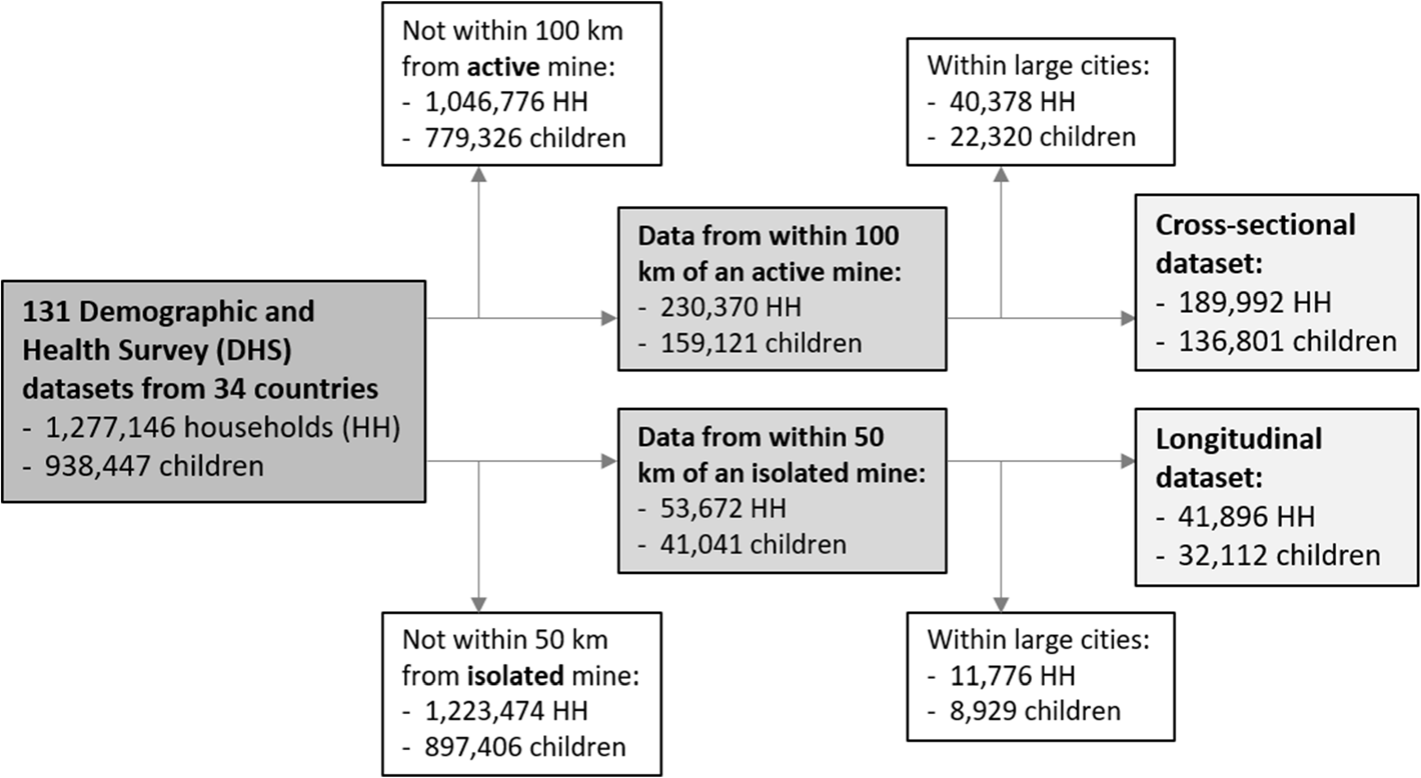
Flow chart of data used for analyses. Only data of households and children located within the proximity of mines and outside the boundaries of large cities were included. Household and child data around active mines were used for the cross-sectional analyses, data from around isolated mines for the longitudinal analyses

Two datasets were created for analysis at the household-level and child-level, respectively (see Fig. 1). For household-level analyses, the combined spatial information (mining activity, location within larger city) was merged with the household recode dataset. For child-level analyses, the child recode dataset was complemented with the spatial information in the DHS cluster dataset and the basic household characteristics in the household recode files.

### Study design

In this study, we first used the cross-sectional datasets to explore the spatial relationships between the mines and water and sanitation infrastructures and associated child health outcomes. In a second step, we created a longitudinal dataset covering households living close to mines that opened during the study period. This dataset was used to estimate the impacts of the development of a mine on these outcomes (see Fig. 1). The household data were used for analyses of the impacts on water and sanitation infrastructures and the child data were used for health outcomes, respectively.

#### Cross-sectional analysis

We computed the distance to the closest active mine for each cluster/household. Clusters outside of a 100 km radius of a mine were excluded. This limit was set to obtain a similar comparison group for the clusters located closer to the mine. Clusters further away may be differently affected by external factors influencing access to water and sanitation infrastructures and child health than clusters closer to the highly impacted areas. Furthermore, survey data obtained before mining activities have started or after closure of the mines were excluded.

#### Longitudinal analysis

Many DHS clusters were located close to multiple mines that opened and closed at different time points. As for these clusters the determination of the start of mining activities in their proximity would be challenging, we focused in the longitudinal analysis on isolated mines. For the longitudinal analysis only data from clusters within a 50 km distance from isolated mines were included, regardless the operational status of the respective mine. This cut-off ensured that the clusters were only located in the proximity of a single mine. Using this data, a pseudo-panel dataset was created comprising of repeated cross-sectional data from the different survey rounds spanning the time frame before and after mine opening.

### Variables

#### Exposure variable

For cross-sectional analyses, the distance between the DHS cluster and the closest active mine during the time of the survey was used. The variable was grouped into 7 categories: ≤5 km, 5–10 km, 10–20 km, 20–30 km, 30–40 km, 40–50 km and 50–100 km. Based on previous studies and field experience of the author team, the impacts of the mines were expected to be limited to the area within a limited travel distance (i.e. around 10 km) [7, 8, 33]. Therefore, this variable was dichotomized in the longitudinal analyses, using ≤ 10 km as impacted area and 10–50 km a comparison area. Furthermore, a variable was created indicating whether the household was surveyed before or after mine opening. The primary exposure variable was the interaction between living in close proximity to the mining project and the mine’s activity status.

#### Outcome variables

##### Water and sanitation infrastructure

All DHS surveys collected data on access to water infrastructures through the same question (“what is the main source of drinking water for members of your household?”). DHS sanitation questions on the other hand have changed slightly over time [34]. Prior to 2003, “what kind of toilet facilities does your household have?” was used. Since then, the question was reworded to “what kind of toilet facility do members of your household usually use?” Furthermore, some countries used additional codes for country-specific types of water and sanitation infrastructure. Nevertheless, the DHS data define broader categories that are applicable to all countries and survey rounds. Therefore, water and sanitation infrastructures were categorized into “basic”, “intermediate” and “modern” [35]. For water sources, surface water and springs were classified as “basic”, well water as “intermediate” and piped water as “modern”. Similarly, no sanitation facility was coded as “basic”, latrines as “intermediate” and flush toilets as “modern”. Other types of water and sanitation infrastructure, such as bottled water, were excluded from analysis.

##### Health outcomes

Based on literature on the impacts of water and sanitation infrastructures on child health, we focused on diarrhea and anthropometry as primary outcomes [25, 26]. All DHS ask caregivers to report diarrheal episodes in the two weeks preceding the survey for children under the age of five years. For a subset of children living in the interviewed households anthropometric data were collected. Children’s height and weight were normalized using the 2006 World Health Organization growth reference standards. A child with a height-for-age z-score below − 2 was considered as stunted. Children with a weight-for-height z-score below − 2 were classified as wasted. Similarly, weight-for-age z-scores below − 2 were considered as underweight.

#### Covariates

Different covariates were integrated in the statistical models, depending on the type of analysis. At the household-level, household wealth was considered as a covariate. The wealth index provided by DHS integrates water and sanitation infrastructure among other household assets for deriving the wealth quintiles. To avoid collinearity with the outcome variables for this study, separate wealth indexes were created. Following the approach proposed in Filmer and Pritchett [36], a principal component analysis with a reduced set of variables (i.e. possession of a car, motorcycle, bicycle, television, radio, fridge, telephone and bank account, access to electricity, wall, flooring and roofing materials, type of cooking fuel, and educational attainment of the head of the household) was conducted. Given that household wealth is both a potential outcome of newly opened mines and a confounding factor, separate models were run with and without the wealth quintile as covariate. Furthermore, stratified analyses were conducted using only the two lower and the two upper wealth quintiles, respectively. Additionally, the number of household members was included as separate covariate at the household-level. At the child-level, age in years (as categorical variable) and gender (female/male) were included in the models. A list of potential covariates were selected a priori and included in the final models based on likelihood ratio tests.

### Statistical analysis

Beyond descriptive statistics for the different outcomes at different distances from active mines, regression models were developed. These differed depending on the outcome and type of analysis. The methodology for the different analyses were developed over the course of the study, without a predefined analysis plan.

#### Cross-sectional descriptive analyses

The cross-sectional dataset was used to describe the average outcome variables at different distances from the mines. To assess the cross-sectional distance associations, multi-level multinomial logistic regression models were used for water and sanitation infrastructure outcomes and multi-level logistic regression models for binary health outcome indicators (see Equation A1 and A2 in Additional file 1). The proximity to active mines was the main exposure of interest. All models included a survey-level random intercept term, accounting for the spatial (between countries) and temporal (between survey rounds) variability. Separate models were run with and without adjustment for the household wealth quintiles. In models for child health outcomes, additional household-level covariates (i.e. household size, access to water and sanitation infrastructures) and child-level covariates (i.e. age and gender) were adjusted for. Additionally, the cross-sectional dataset was used to describe cross-country differences in the associations between mining and the different outcomes. Distances between the households and the mines of up to 10 km were considered as impacted, while households located between 20 and 100 km were used as comparison. The regression models adjusting for household-level and child-level (for child health outcomes only) covariates were used for this analysis.

#### Longitudinal analyses

Our main impact analysis explored a quasi-experimental difference-in-difference (DiD) design. The repeated random samples of households in the DHS allowed us to observe infrastructure and child outcomes in close proximity to mines and in neighboring areas over time. If the location and timing of mine openings are not systematically correlated with other factors affecting our outcomes of interest, identical trends in outcome variables before and after the mine opening would be expected in the absence of a causal change induced by the mine itself [37]. While we could not directly verify this assumption of common trends, we can test equality of trends prior to the mine opening empirically. By comparing trends in mining areas to nearby locations, we could account for factors specific to the study site or survey methodology (e.g. urbanization, seasonality). The equations in Additional file 1 show the estimated equations for the household for child-level analyses. Our main variable of interest was the interaction term between being located in close proximity (≤ 10 km) a mine and the mine being active. We included a mine-level random intercept term instead of the survey-level term to account for the pseudo-panel structure of the data using repeated measurements around the different mines.

Multi-level multinomial logistic regression models (for water and sanitation infrastructure) were estimated using the generalized structural equation modelling suite in StataSE version 16 (StataCorp LLP, College Station, TX, USA). R version 3.5.1 [38] was used for running the multi-level logistic regression models with binary outcomes (for child health outcomes). Statistics were reported with their associated 95 % confidence intervals (CI), where applicable.

## Results

In total, data from 1,277,146 households in 34 countries were included in the study (Fig. 1). The countries included Angola, Burkina Faso, Benin, Burundi, Cameroon, Central African Republic, Chad, Comoros, Côte d’Ivoire, Democratic Republic of the Congo, Eswatini, Ethiopia, Gambia, Ghana, Guinea, Kenya, Liberia, Lesotho, Madagascar, Malawi, Mali, Mozambique, Namibia, Niger, Nigeria, Rwanda, Senegal, Sierra Leone, South Africa, Tanzania, Togo, Uganda, Zambia, and Zimbabwe. Household and geographical information was available for 938,447 children. Overall, there were 2,016 mines in the mining dataset. For 711 mines, information on operational activities were reported between 1980 and 2019. After selection of clusters located within 100 km of active mines and exclusion of data from larger cities, 189,992 households and 136,801 children from 27 countries were included in the cross-sectional analyses. In Benin, Cameroon, Central African Republic, Chad, Comoros, Rwanda and Togo no active mine with DHS clusters was present. Of the 711 mines with activity status information, 52 were more than 100 km away from the next mine and were therefore included to create the pseudo-panels for longitudinal analyses. In the Côte d’Ivoire, Eswatini, Lesotho and Senegal the mines were not isolated and hence, data from these countries not included in the longitudinal datasets. The final dataset, consisting of repeated cross-sectional survey data around these isolated mines, comprised 41,896 households and 32,112 children from 23 countries. Basic descriptive statistics for household and child health indicators for the four datasets are presented in Table 1. Changes in the percentage of households categorized as wealthy or poor are shown in Additional file 2. In areas close to the mines, the percentage of poor households decreased substantially after mine opening, while it remained comparably constant in comparison areas.

**Table 1:** Descriptive statistics for different household and child indicators

	Household data		Child data	
	Cross-sectional dataset (*N*=189 992)	Longitudinal dataset (*N*=41 896)	Cross-sectional dataset (*N*=136 801)	Longitudinal dataset (*N*=32 112)
**Distance to mine**				
≤5 km	2 893 (1.5%)	n.a.	1 722 (1.3%)	n.a.
5-10 km	5 654 (3.0%)	n.a.	3 505 (2.6%)	n.a.
10-20 km	13 527 (7.1%)	n.a.	8 902 (6.5%)	n.a.
20-30 km	15 927 (8.4%)	n.a.	11 539 (8.4%)	n.a.
30-40 km	20 581 (10.8%)	n.a.	14 177 (10.4%)	n.a.
40-50 km	21 341 (11.2%)	n.a.	15 223 (11.1%)	n.a.
50-100 km	110 069 (57.9%)	n.a.	81 733 (59.7%)	n.a.
**Mine close (≤10 km)**	n.a.	2 857 (6.8%)	n.a.	1 894 (5.9%)
**Mine active**	n.a.	17 805 (45.7%)	n.a.	12 738 (42.5%)
**Water infrastructures**				
Basic (surface water)	35 802 (19.1%)	11 052 (26.7%)	28 484 (21.1%)	8 794 (27.6%)
Intermediate (well)	85 141 (45.4%)	19 130 (46.2%)	68 828 (50.9%)	15 549 (48.8%)
Modern (piped/tap)	61 967 (33.1%)	10 812 (26.1%)	35 940 (26.6%)	7 390 (23.2%)
**Sanitation infrastructures**				
Basic (no facility)	50 061 (26.8%)	12 859 (31.3%)	40 037 (29.8%)	10 883 (34.4%)
Intermediate (latrine)	112 190 (60.2%)	25 461 (61.9%)	82 219 (61.2%)	19 363 (61.2%)
Modern (flush toilet)	24 233 (13.0%)	2 807 (6.8%)	12 152 (9.0%)	1 402 (4.4%)
**Household size (median)**	4	5	6	6
**Wealth quintile**				
Poorest	38 064 (20.0%)	8 714 (20.8%)	27 586 (20.6%)	6 714 (20.9%)
Poor	41 949 (22.1%)	8 381 (20.0%)	30 730 (22.9%)	6 604 (20.6%)
Middle	42 330 (22.3%)	9 067 (21.7%)	31 607 (23.6%)	7 144 (22.3%)
Rich	38 700 (20.4%)	9 037 (21.6%)	27 296 (20.4%)	7 189 (22.4%)
Richest	28 949 (15.2%)	6 609 (15.8%)	16 792 (12.5%)	4 433 (13.8%)
**Stunted**	n.a.	n.a.	22 402 (34.0%)	5 878 (40.8%)
**Wasted**	n.a.	n.a.	4 697 (7.2%)	1 224 (8.6%)
**Underweight**			10 918 (16.3%)	3 211 (22.0%)
**Diarrheal episode**^**a**^	n.a.	n.a.	16 121 (15.2%)	4 585 (18.4%)
**Age (mean/years)**	n.a.	n.a.	1.9	1.9
**Female**	n.a.	n.a.	67 876 (49.6%)	15 866 (49.4%)

Data from 131 Demographic and Health Surveys from 34 sub-Saharan Africa collected between 1990 and 2019 within 100 km from active mines (cross-sectional datasets) or within 50 km from isolated mines (longitudinal dataset) were included. Only households or children with non-missing data were used as denominators for the percentages

*n.a.* not applicable

^a^Presence of a diarrheal episode during the two weeks prior to the survey

### Access to water and sanitation infrastructures

#### Associations between distance to mine and water and sanitation infrastructures

**Fig. 2:**
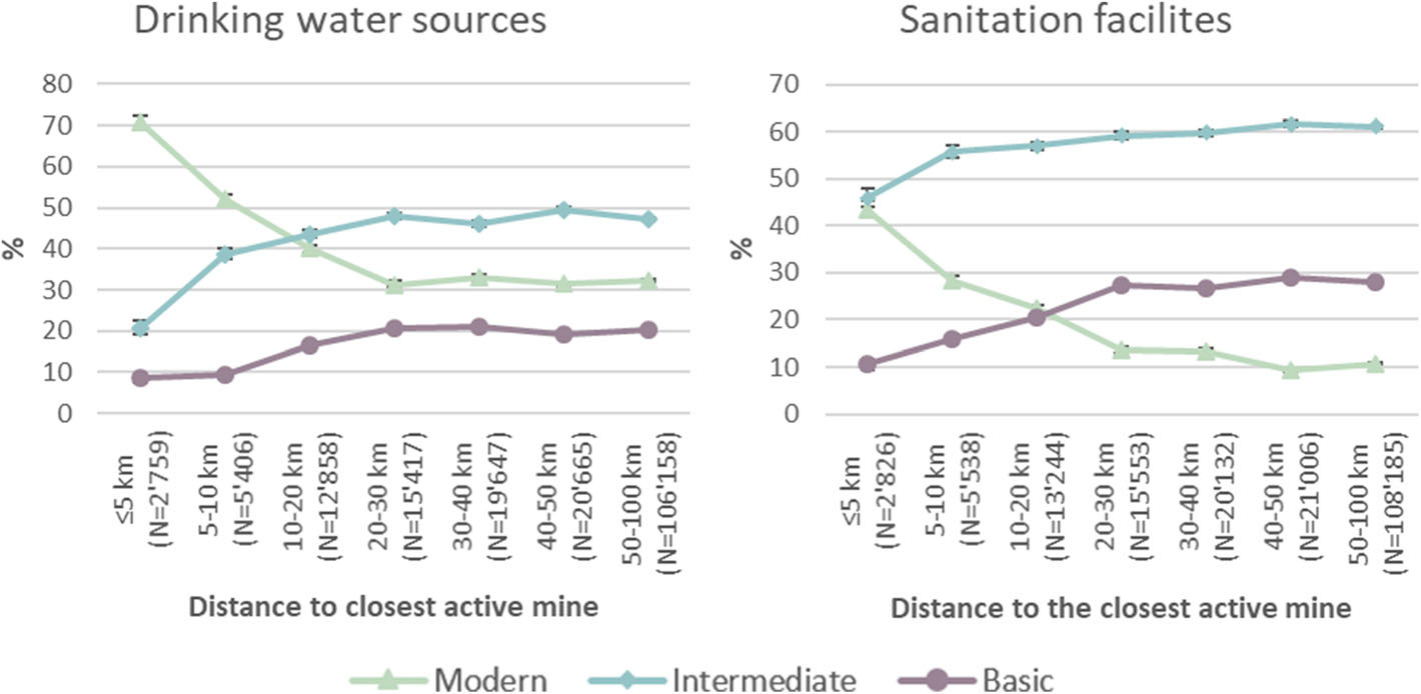
Percentage of drinking water sources and sanitation facilities by distance to the closest active mine. Error bars show 95 % confidence intervals

Figure 2 shows average access to water and sanitation infrastructure by distance to active mines. The share of households having access to modern drinking water sources was almost 40 % points higher close to the mines (i.e. up to 5 km) than outside a 20 km radius. In contrast, households located further away relied more often on water from wells (intermediate) or surface water sources (basic). These trends were seen up to a distance of 20 km.

Similarly, there was also a trend towards more modern sanitation infrastructures closer to the mines. For example, while 43.5 %, 95 % CI: 41.7 – 45.4 %, of households located up to 5 km from an active mine had access to a modern sanitation facility, only 10.8 %, 95 % CI: 10.6 – 11.0 %, of households at a distance between 50 and 100 km had access to such facilities. On the other hand, basic infrastructures were more widespread in the areas located further away from the mines.

These trends were also seen in the results from the regression models (see Additional files 3, 4 and 5). The associations between the proximity to mines and access to modern water and sanitation infrastructures were significant up to a distance of 20 km from the mines.

#### Impact of mine opening on access to water and sanitation infrastructures

**Fig. 3:**
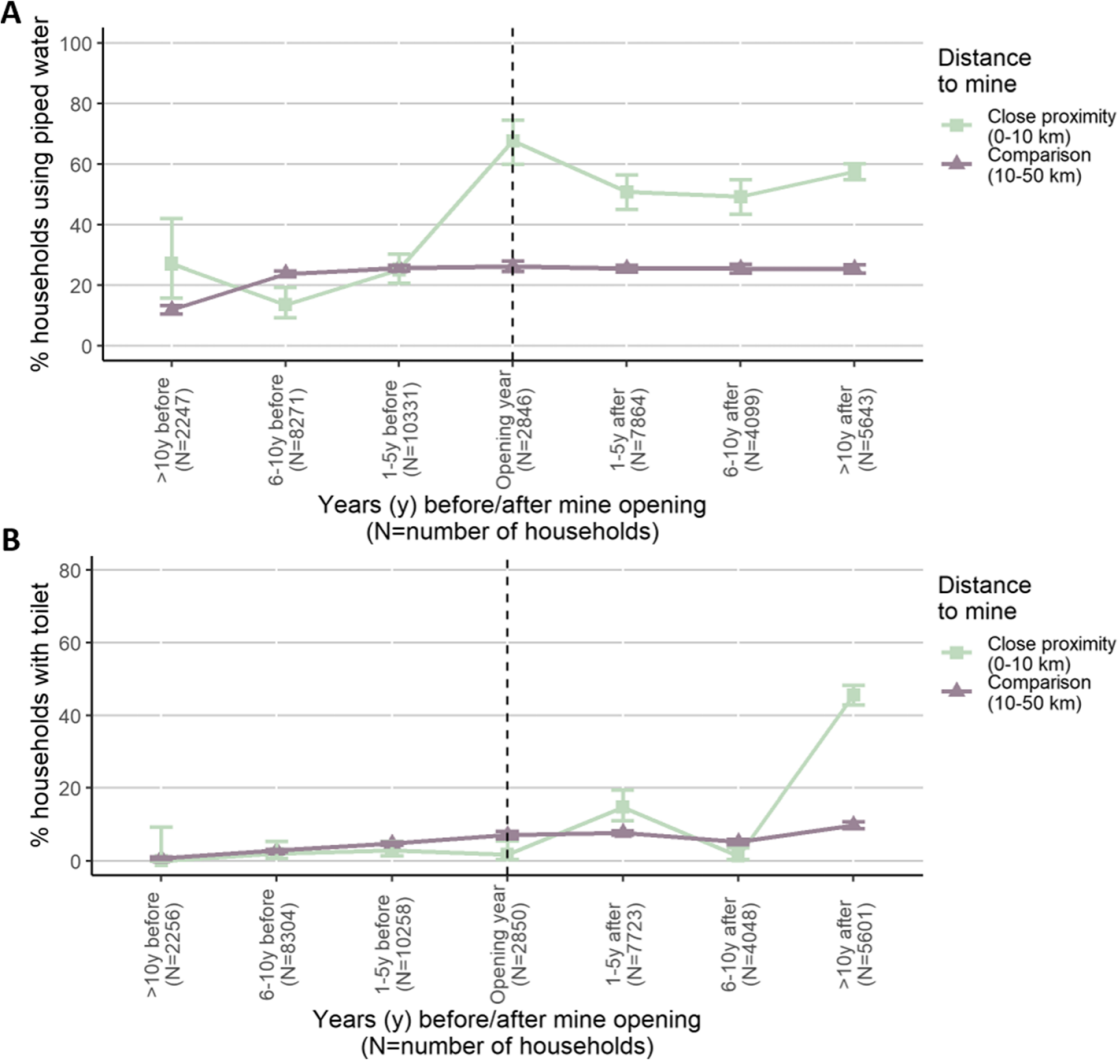
Percentage of households having access to piped water (panel **A**) and flush toilets (panel **B**). The x-axis shows the time period of the survey relative to the opening year of the mine. Separate graphs are shown for each distance category (≤ 10 km vs. 10–50 km from the mine). Error bars show 95 % confidence intervals

Figure 3 shows the change in access to water and sanitation infrastructures relative to the opening year of the mine stratified by distance between the household and the mine. Shortly after mine opening, the share of households in the proximity (i.e. at a ≤ 10 km distance) using modern drinking water sources increased sharply, while for sanitation infrastructures, marked changes occur after 10 years or later. In households located further away (i.e. between 10 and 50 km from the mine), the improvements in access to modern water and sanitation facilities over time were less pronounced.

**Fig. 4:**
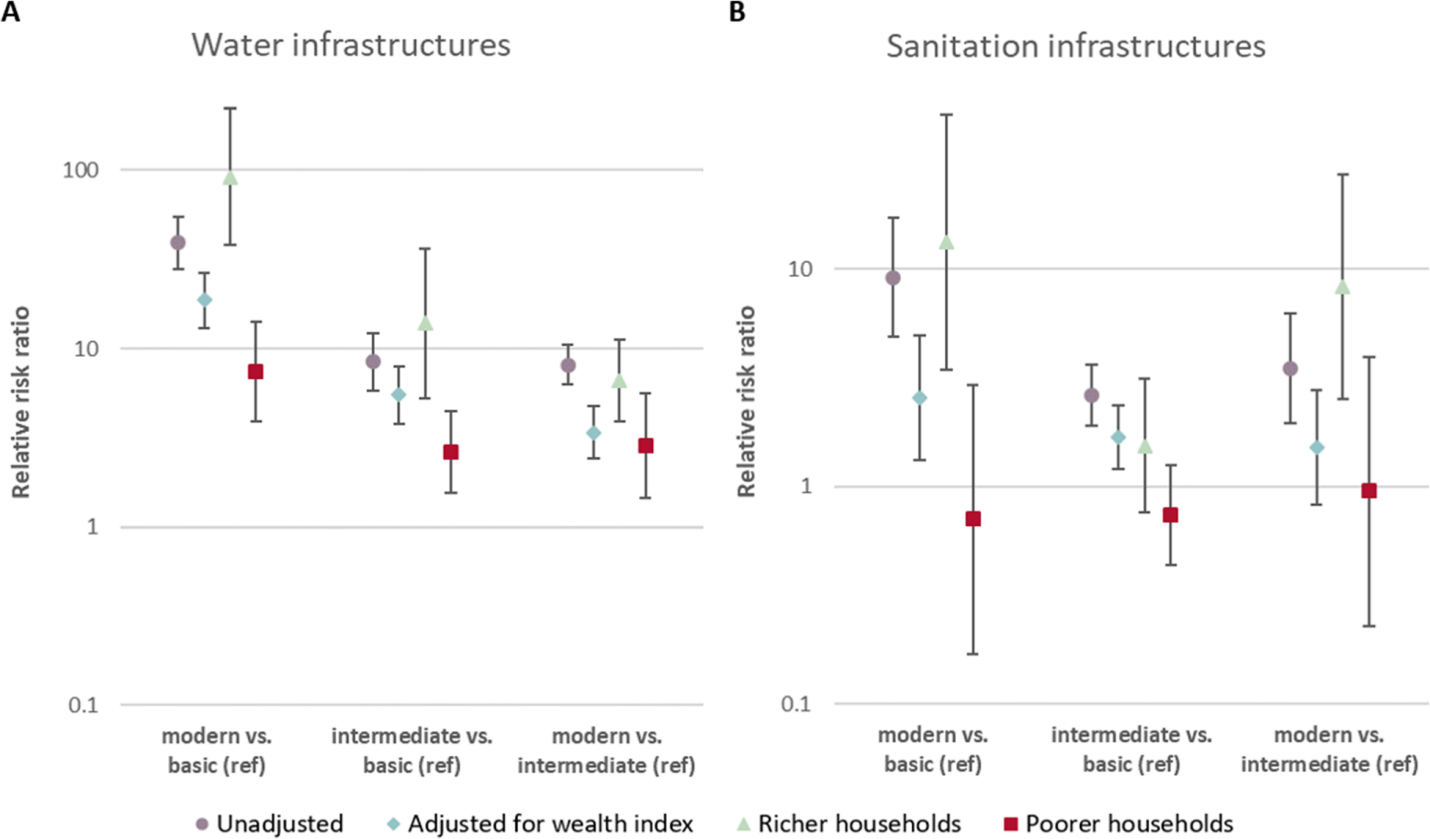
Estimates of the relative risk ratios (RRR) for the interaction of mining activity (before vs. after mine opening) and proximity to the mine (≤ 10 km vs. 10–50 km) on access to water (panel **A**) and sanitation (panel **B**) infrastructures using the longitudinal household dataset. The RRR and their corresponding 95 % confidence intervals were derived using the generalized structural equation modelling suite in Stata and plotted on the log-scale

The regression analyses using the longitudinal dataset compared the impact of the proximity to mines on water and sanitation infrastructures before and after mine opening (see Fig. 4; Table 2). In line with the results from the cross-sectional analyses, the opening of mining projects had strong and positive impacts on access to modern water infrastructures of the households in their proximity. More specifically, the change in the access to modern water infrastructures (compared to basic infrastructures) upon mine opening was 18.6-times higher in households near mines than households located in comparison areas (adjusted relative risk ratio (aRRR): 18.60, 95 % CI: 13.08–26.46). These positive effects were more pronounced among the wealthier households compared to the poorer households. Particularly for the wealthier households, the use of basic water infrastructures was very low after mine opening, leading to high aRRR estimates. Furthermore, stronger associations were found when excluding data collected during a potential transition phase two years before and two years after mine opening (see Additional file 6).

Also for the sanitation categories, the establishment of a mining project had a positive impact as seen in the longitudinal analyses (Fig. 4; Table 2). Overall, the households closer to the mine had increased access to more modern sanitation facilities after the mines became active. Stratified analyses revealed that this effect was only seen among the wealthier households (RRR comparing modern vs. basic sanitation infrastructures: 13.20, 95 % CI: 3.43–50.89) but not among the poorer households (RRR comparing modern vs. basic sanitation infrastructures: 0.71, 95 % CI: 0.17–2.94).

**Table 2:** Relative risk ratios (RRR) for the effect of the interaction between mining activity (before vs. after mine opening) and proximity to the mine (≤10 km vs. 10-50 km) on access to water and sanitation infrastructures using the longitudinal household dataset

	RRR (95%CI) for interaction close proximity*active			
	Crude^**a**^ (N_water_=38,088) (N_sanitation_=38,190)	Adjusted^**b**^ (N_water_=38,088) (N_sanitation_=38,190)	Rich^a,**c**^ (N_water_=14,099) (N_sanitation_=14,264)	Poor^a**,c**^ (N_water_=15,605) (N_sanitation_=15,563)
Water: modern vs. basic (ref)	39.25 (28.02 - 54.97)**	18.60 (13.08 - 26.46)**	91.73 (38.09 - 220.87)**	7.46 (3.92 - 14.16)**
Water: intermediate vs. basic (ref)	8.43 (5.84 - 12.17)**	5.48 (3.79 - 7.92)**	13.83 (5.28 - 36.23)**	2.63 (1.55 - 4.47)**
Water: modern vs. intermediate (ref)	8.12 (6.29 - 10.47)**	3.39 (2.41 - 4.79)**	6.61 (3.88 - 11.24)**	2.84 (1.44 - 5.58)*
Sanitation: modern vs. basic (ref)	9.15 (4.91 - 17.04)**	2.56 (1.32 - 4.99)**	13.20 (3.43 - 50.89)**	0.71 (0.17 - 2.94)
Sanitation: interme-diate vs. basic (ref)	2.63 (1.91 - 3.62)**	1.69 (1.21 - 2.37)*	1.54 (0.76 - 3.14)	0.74 (0.44 - 1.26)
Sanitation: modern vs. intermediate (ref)	3.47 (1.95 - 6.20)**	1.52 (0.83 - 2.78)	8.28 (2.54 - 26.97)**	0.96 (0.23 - 3.92)

The estimates and their corresponding 95% confidence intervals (95% CI) were derived using the generalized structural equation modelling suite in Stata

^a^mine-level random intercept only

^b^adjusted for household wealth quintile

^c^stratified analyses using only data from the two lower wealth quintiles (poorer households) and the two upper wealth quintiles (wealthier households), respectively

* *p* < 0.05; ** *p*<0.001

### Child nutrition and diarrhea

#### Associations between distance to mine and child health outcomes

**Fig. 5:**
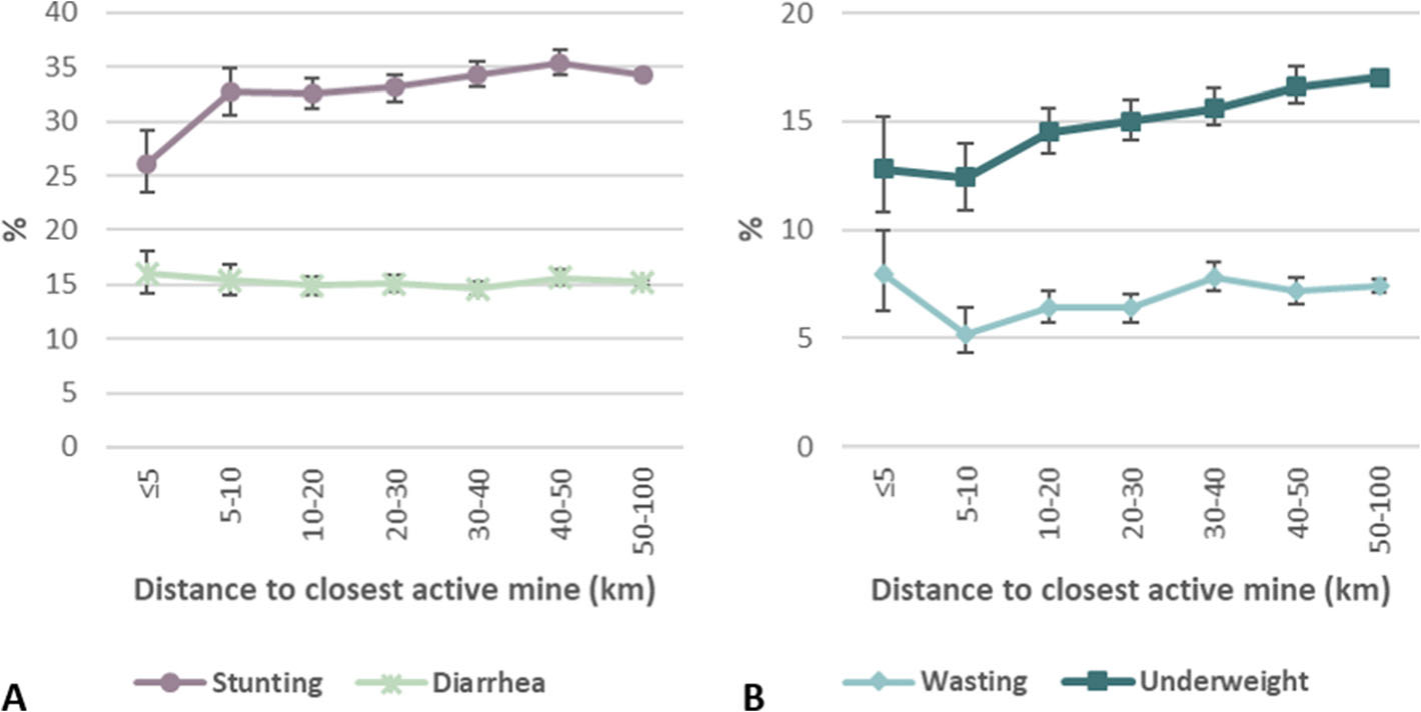
Prevalence of anthropometric indicators and 2-week diarrheal prevalence among children under five years near mines. Results are stratified by distance to the closest active mine. Error bars show 95 % confidence intervals

Figure 5 shows the differences in child health indicators at different distances from the mine Stunting was less common within a 5 km radius from the mines (26.1 %, 95 % CI: 23.4–29.1 %) compared to children living further away (e.g. 50–100 km: 34.3 %, 95 % CI: 33.8–34.7 %). A slight increasing trend in the 2-week prevalence of diarrhea among children under five years was seen closer to the mines. Wasting prevalence ranged from 6.4 to 8.0 %. Highest percentages were observed closest to the mines. For underweight, the lowest percentages of around 12.5 % were seen within a 10 km radius from the mines. Further away, underweight rates increased up to 17 % at a distance of 50–100 km.

In the regression models, the associations between mining projects and child health outcomes were less clearly seen (see Additional files 7 and 8). In the models adjusted for child-level covariates, an increase in child wasting was seen in proximity to the mines, while for stunting a reduction in the odds at this distance was observed.

#### Impact of mine opening on child health outcomes

**Fig. 6:**
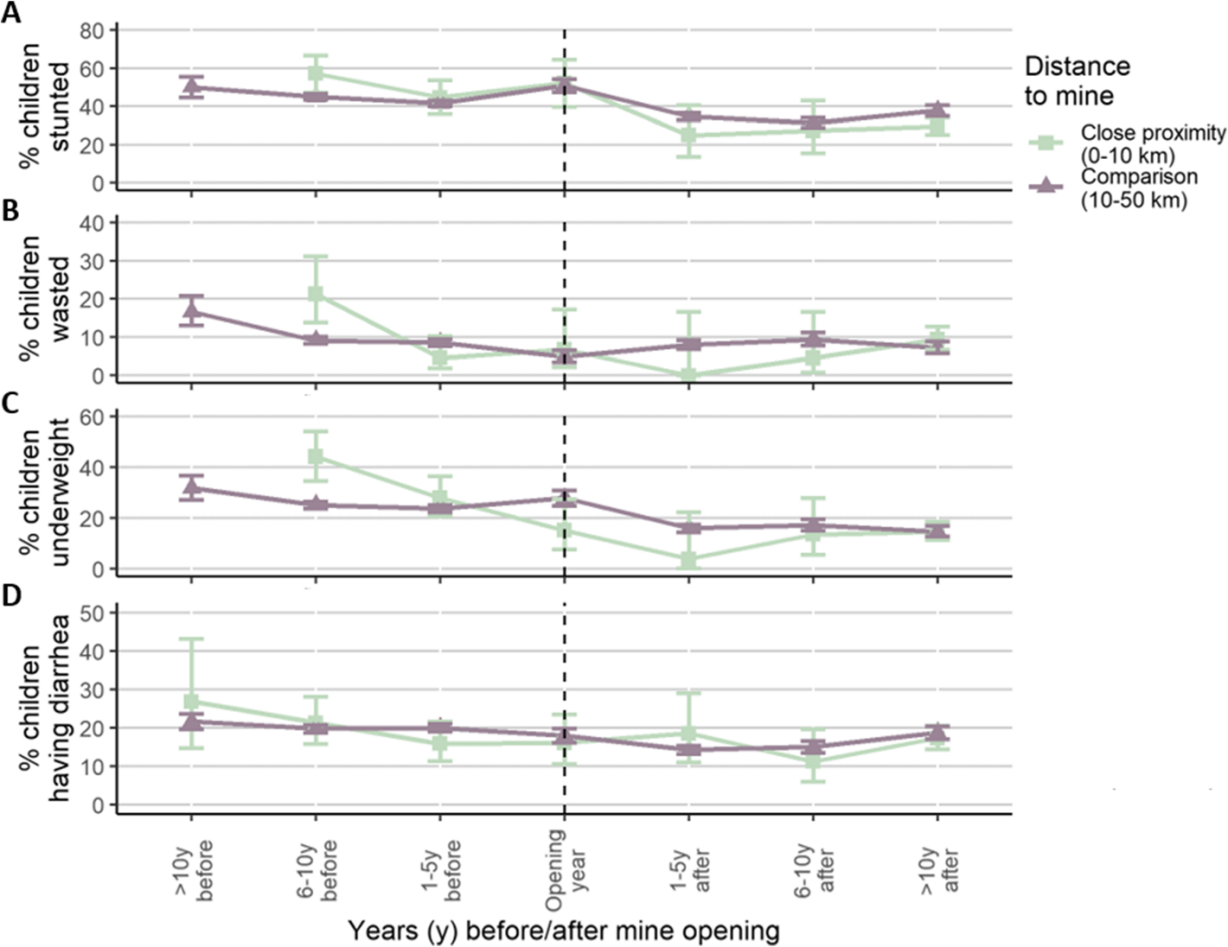
Percentage of children stunted (panel **A**), wasted (panel **B**), underweight (panel **C**) and suffering from diarrhea in the two weeks prior to the survey (panel **D**). The x-axis shows the time period of the survey relative to the opening year of the mine. Separate graphs are shown for each distance category (≤ 10 km vs. 10–50 km from the mine). Error bars show 95 % confidence intervals. No anthropometric data were available in the close areas from more than 10 years before mine opening

Figure 6 summarizes the estimated mining impact on child health outcomes. On average, child health outcomes in areas close to mines were substantially worse in the surveys conducted more than 5 years prior to the mine opening, but looked relatively similar in the 5 years before the mine became active. Stunting, wasting and underweight prevalences declined more rapidly in areas close to mines after mining activities commence, with fading differences over time. Diarrhea prevalences were similar before and after mining operations were launched.

**Fig. 7:**
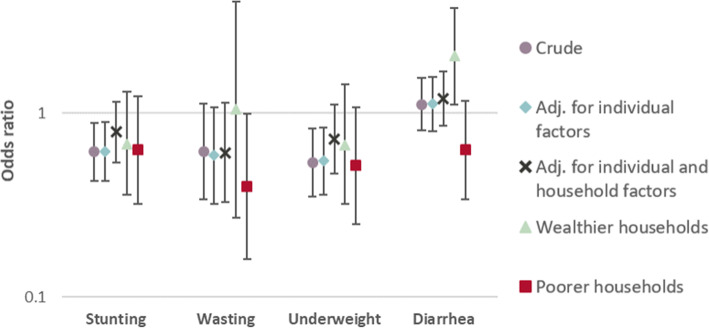
Estimates of the odds ratios (OR) and corresponding 95 % confidence intervals for the interaction of mining activity (before vs. after mine opening) and proximity to the mine (≤ 10 km vs. 10–50 km) on child health indicators using the longitudinal dataset on children under 5 years

The results from the regression analyses drawing from the longitudinal dataset comprising of repeated cross-sectional survey data are shown in Fig. 7; Table 3. Adjusting for child-level factors, the opening of a mine reduced the odds of stunting and underweight by 38 and 45 %, respectively relative to the comparison areas (adjusted odds ratio (aOR) for stunting: 0.62, 95 % CI: 0.43–0.90; aOR for underweight: 0.55, 95 % CI: 0.36–0.84). For wasting, the interaction term between mining proximity and activity was not statistically significant, although when excluding data from a potential transition period, a significant reduction in wasting was observed (see Additional file 9). Furthermore, among children in poorer households, a reduction in the odds of wasting was seen using the complete dataset (aOR: 0.40, 95 % CI: 0.16–0.99). For diarrhea, children in better-off households in close proximity to active mines experienced increased odds of sickness relative to control areas (aOR: 2.05, 1.11–3.76). Other associations were not statistically significant.

**Table 3:** Odds ratios (OR) and 95% confidence intervals (95% CI) for the effect of the interaction between mining activity (before vs. after mine opening) and proximity to the mine (≤10 km vs. 10-50 km) on childhood health outcomes using the pseudo-panel household dataset

	OR (95%CI) for interaction close proximity*active				
	Crude model^a^	Adj. for ind. factors^b^	Adj. for ind. and HH factors^c^	Wealthier HH only^bd^	Poorer HH only^bd^
Stunting	0.62 (0.43 - 0.89)*	0.62 (0.43 - 0.90)*	0.79 (0.54 - 1.16)	0.68 (0.36 - 1.31)	0.63 (0.32 - 1.24)
Wasting	0.62 (0.34 - 1.13)	0.59 (0.32 - 1.08)	0.61 (0.33 - 1.14)	1.05 (0.27 - 4.06)	0.40 (0.16 - 0.99)*
Underweight	0.54 (0.35 - 0.83)*	0.55 (0.36 - 0.84)*	0.72 (0.47 - 1.11)	0.67 (0.32 - 1.43)	0.52 (0.25 - 1.08)
Diarrhea	1.12 (0.81 - 1.56)	1.13 (0.80 - 1.58)	1.20 (0.85 - 1.69)	2.05 (1.11 - 3.76)*	0.63 (0.34 - 1.17)

^a^mine-level random intercept only

^b^adjusted for individual-level factors (child age and sex)

^c^adjusted for individual and household-level factors (wealth, access to water and sanitation, household size)

^d^stratified analyses using only data from the two lower wealth quintiles (poorer households) and the two upper wealth quintiles (wealthier households), respectively

*p < 0.05; ** *p*<0.001

### Cross-country differences

**Fig. 8:**
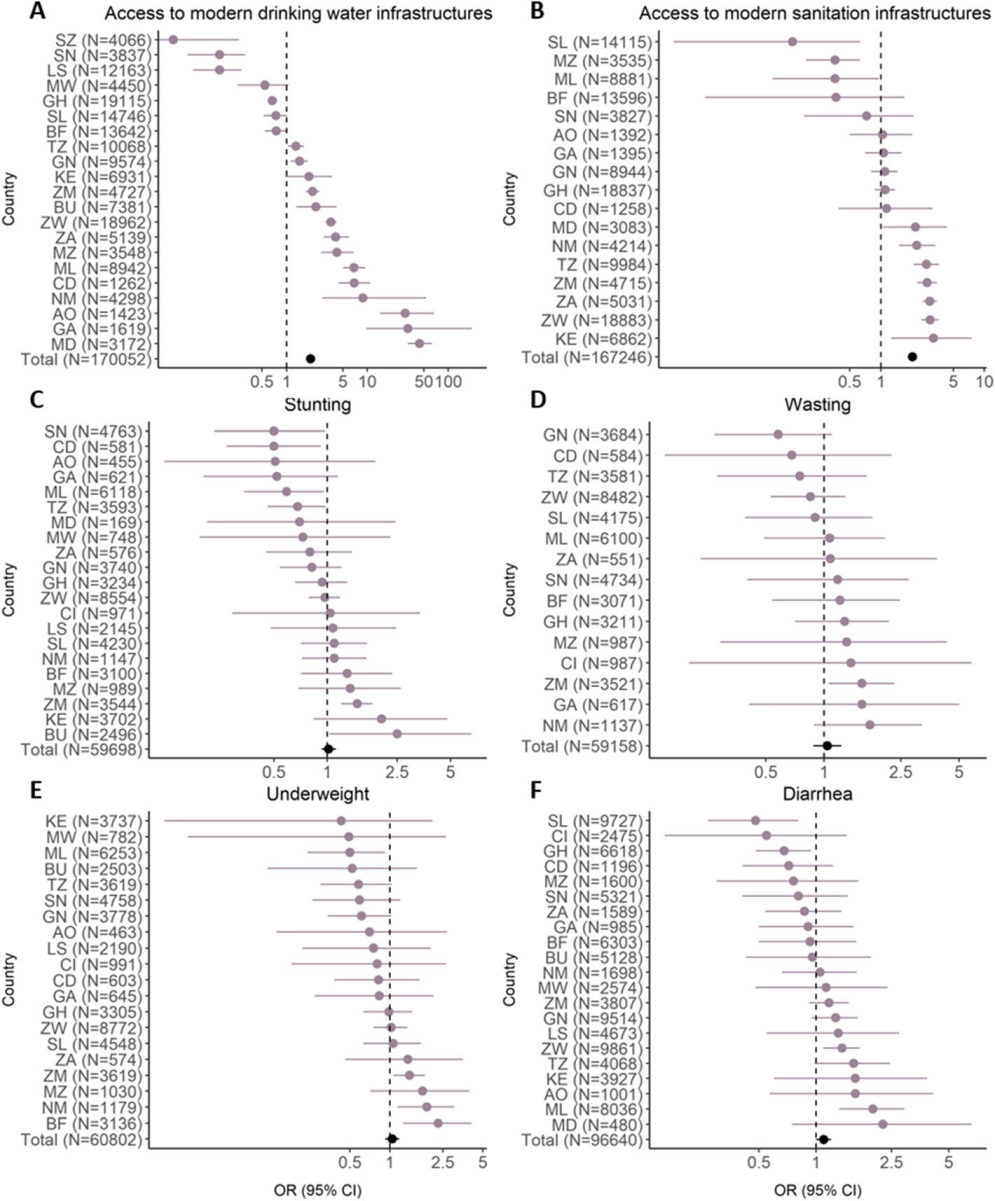
Country-level analysis of the impact of mines on water and sanitation infrastructures and child health. The forest plot shows the odds ratios (OR) and corresponding 95 % confidence intervals (95 % CI) for the association between distance to a mine (≤ 10 km vs. 20–100 km) and modern water (panel **A**) and sanitation (panel **B**) infrastructures, stunting (panel **C**), wasting (panel **D**), underweight (panel **E**) and diarrhea (panel **F**) among children under 5 years stratified by country. Some countries were excluded due to the low number of households in the respective categories close to the mines. The overall OR for all countries in the dataset is shown in black color. AO = Angola; BF = Burkina Faso; BU = Burundi; CD = Democratic Republic of Congo; CI = Ivory Coast; GA = Gabon; GH = Ghana; GN = Guinea; KE = Kenya; LS = Lesotho; MD = Madagascar; ML = Mali; MW = Malawi; MZ = Mozambique; NM = Namibia; SL = Sierra Leone; SN = Senegal; SZ = Eswatini; TZ = Tanzania; ZA = South Africa; ZM = Zambia; ZW = Zimbabwe

Figure 8 shows the associations between the distance to the mines and the different types of water and sanitation infrastructures as well as associated health outcomes by country. Large differences in the point estimates between the different countries were seen for all comparisons. For the comparisons modern vs. basic of both water and sanitation infrastructures the majority of countries showed positive associations with mining projects. Still, in some countries households close to mines were less likely to have access to modern water and sanitation infrastructures. Also for the health outcomes, there were marked differences in the OR between the countries. However, only in few countries statistically significant associations were seen. For example, a reduction of stunting rates was seen in Senegal, Mali, Tanzania and the Democratic Republic of Congo. On the other hand, increased odds for stunting close to the mines were seen in Zambia and Burundi. Some countries had to be excluded from the analyses due to the low case numbers in close proximity to mines, particularly for wasting.

## Discussion

In the present study, the largest dataset integrating household and child health data from 34 sub-Saharan African countries together with a comprehensive list of mines was used for comparing trends in household infrastructure and child health in areas close to mines as well as neighboring areas over time. The results indicate that access to modern water infrastructures improved rapidly after mine opening, while positive changes in sanitation infrastructures started to manifest after 10 years of operation. Some improvements in child health outcomes were seen, such as in stunting and underweight prevalences. No clear trends in wasting or diarrhea were observed. Changes in household wealth seemed to play an important role in determining the distribution of benefits. Furthermore, large cross-country differences were observed, both for the associations between mining and water and sanitation infrastructures and the child health indicators. In summary, despite the positive impacts of mines on water and sanitation infrastructures, related child health indicators in mining communities only partially improved. These findings suggest that factors other than water and sanitation infrastructures also affect child health in mining communities.

The trends in access to sanitation found in the present study are in line with another study focusing on data from Mali [21]. Although their estimates were not significant, they also found similar trends with better infrastructures close to the mines but decreased access to modern facilities at intermediate distances (30–60 km). On the other hand, Ouoba et al. found no effect on water and sanitation infrastructure around mines in Burkina Faso [39], and Polat and colleagues even found negative impacts [20]. Potential reasons for the different results could be that the former study analyzed data at a relatively coarse spatial resolution (regional-level) while the latter used data at a much larger distance from the mines (i.e. up to 250 km) as comparison group and did not exclude larger cities. This may potentially have led to more urban comparison areas than the predominantly rural mining sites.

The positive impacts of mining projects on water and sanitation infrastructures were less seen among the poorer households, particularly for access to modern sanitation. Poor households in mining areas potentially constitute of a selected group of people that remains poor despite overall economic development or include migrants that settle in informal dwellings with particularly low access to water and sanitation infrastructures [18, 40, 41]. Hence, the poor living in mining areas may differ from people living in poor households elsewhere [40]. As a result, despite the overall improvements in infrastructures in mining areas, informal settlements remain underserved. Furthermore, access to modern sanitation infrastructures improved at a slower pace than water infrastructures. This may indicate that while investments in local water infrastructures are often part of mining project’s corporate social responsibility programs, investments in sanitation infrastructures are less common [5]. Hence, improvements in sanitation infrastructures are only seen later, potentially as a result of rising household wealth in mining communities. This is in accordance with an analysis of impact assessment reports of mining projects in sub-Saharan Africa that revealed that efforts to improve access to sanitation infrastructures were less frequently included in mitigation plans than for water infrastructures [42]. The large variations in associations found across the different studies, as well as across and within countries, underlines the importance of assessing local-level trends around mining projects in order to promote an equitable distribution of foster potential positive impacts on sustainable development in mining communities [43]. Identification of vulnerable population sub-groups in mining communities in the management of mining projects would contribute to a more equitable distribution of their benefits [44].

As water and sanitation infrastructures are important contributors to health and wellbeing, progress towards SDG 6 could directly or indirectly also promote SDG 3 (“good health and wellbeing”) [45]. Quantitative studies indicate that improvements in water and sanitation infrastructures have been shown to reduce child mortality, malnutrition and diarrhea [22, 46]. However, in our study improvements in child health indicators were less evident. For example, diarrheal diseases did not decrease in mining areas as seen in other studies [20, 21, 28]. As our results suggest, the associations between the proximity to a mine and childhood diarrhea varies by country, which could also explain the ambiguous findings of case studies focusing on individual countries [21]. Further, these variations may also be the reason for the absence of significant associations when using the multi-country dataset. Another explanation for both the absence of an association and the variations between countries may be that diarrhea episodes were self-reported by the mothers. Self-reported diarrhea is subject to recall and reporting bias and the concept and terminology of diarrheal diseases varies between geographical regions [47]. Research has shown that diarrheal diseases are more often recognized and reported by literate mothers or by care-givers living in households with access to improved water sources [48]. Although these effects are directly or indirectly (through the household wealth index) adjusted for, it is possible that this source of bias has concealed the beneficial effects of the improved access to modern water and sanitation infrastructure in mining areas. However, it is also possible that other pathways of diarrheal infections are affected by the establishment of mining projects, such as hampered water quality, increased population density or changes in transport and storage practices of drinking water [15, 49]. More in-depth research is needed to elucidate what factors contribute to childhood diarrhea in mining areas, ideally drawing from longitudinal data of cohorts.

Anthropometric data on the other hand are less prone to bias, as they are measured by trained investigators according to standardized procedures. Indeed, in our study we found reduced rates of childhood stunting and underweight. Contrarily, we find higher rates of acute malnutrition (wasting) in the proximity (i.e. ≤5 km) to mining projects in the cross-sectional dataset. However, these associations were not seen at larger distances from the mines. Similarly to our findings, von der Goltz et al. found reduced stunting rates in a large-scale analysis using a similar dataset around mines in developing countries [8]. Further, a study in Zambia has found similar results, although these results were only marginally significant [27]. On the other hand, higher levels of stunting among children under five years in Tanzania but lower rates in Mali were found in another study [20]. Studies on wasting and underweight rates in mining areas are rare and evidence is inconclusive. In a study in Zambia no marked differences were seen for wasting or underweight [28], while in Tanzania and Mali, lower rates of underweight were observed in areas close to mines [20]. The overall lower chronic but higher acute malnutrition rates found in the present study could potentially be explained by land use and life style changes around large mines, away from subsistence agriculture [50, 51]. This may decrease the quality of the children’s diet and decrease household’s resilience to short-term fluctuations in food availability [50]. However, this hypothesis merits further investigation in agricultural practices and changes in dietary patterns in mining regions.

The overall positive impacts on water and sanitation infrastructures underline the potential of mining projects to promote the 2030 Agenda for Sustainable Development [52]. However, for these impacts to improve community health as well as for ensuring an equitable distribution of these benefits, appropriate regulatory and policy frameworks need to be in place [44, 53]. Health impact assessment or inclusion of health in other forms of impact assessment can be a suitable tool for addressing health issues during the licensing process and subsequent management of the risks and opportunities of mining projects [54].

Our results are subject to a set of limitations. First, the focus was set only on industrial mines and did not differentiate between the types of commodities extracted. Artisanal and small-scale mines that are often located in proximity to larger mines can by themselves attract a large number of people and have a series of environmental, social and health impacts [55, 56]. Second, in our analyses we considered the mine opening year as the time impacts are expected. However, during the construction phase, often characterized by a peak in the demand of workforce, potential impacts could already start to manifest [57]. The sensitivity analyses, masking out data during a theoretical transition period before and after mine opening, addressed this shortfall. Yet, accurate data on construction duration would be needed to test for any differential impacts during the different mining phases. Third, the cross-sectional nature of the data does not allow for assessing causation of the associations found. Fourth, there is potential for unmeasured confounding from factors inherent to mining areas, such as increased levels of urbanization. To address this, larger cities were excluded from analyses. Additionally, population density estimates were tested as covariates in the regression models. However, they did not improve model fit. Fourth, survey data can be prone to recall and reporting bias. Furthermore, the DHS questionnaires have slightly changed over time and local understanding of the questions may vary. Hence, only a rough classification of water and sanitation infrastructures was possible, clustering some infrastructures together that would be classified into different categories if the established water and sanitation service ladders were used [35, 58, 59]. Lastly, inaccuracy of GPS data, both the systematic errors introduced in the DHS data and the random errors in the mining projects dataset, could have diluted our results and reduce statistical power [60]. However, the resulting bias is expected to be non-differential.

## Conclusions

Our results suggest that mining projects can have a positive contribution to the work towards “universal access to improved and reliable water and sanitation for all” (SDG 6) and improved child health (SDG 3). Given that the risks and benefits of mines vary strongly between countries and across socio-economic strata, it is crucial that health is adequately addressed in the licensing process of mining projects. A rigorous assessment and management of potential health impacts can not only ensure that benefits are equitably distributed throughout all social strata, but also to expand the potential health benefits of the mining sector to impacted communities. With the right policy framework in place, mining projects have substantial potential to be an important contributor in the work towards achieving the goals of the 2030 Agenda for Sustainable Development, helping to achieve the ultimate goal to “leave no one behind”.

## Supplementary Information

**Additional file 1.** Equations.

**Additional file 2.** Percentage of households being classified as wealthy and poor.

**Additional file 3.** Spatial trend of the relative risk ratios for the association between the distance to the closest mine and water and sanitation infrastructures.

**Additional file 4.** Results from the regression models for the association between distance to mine and water infrastructures.

**Additional file 5.** Results from the regression models for the association between distance to mine and sanitation infrastructures.

**Additional file 6.** Sensitivity analysis for the interaction effect on water and sanitation infrastructure.

**Additional file 7.** Spatial trend of the odds ratios for the association between the distance to the closest mine and stunting, wasting, underweight and 2-week diarrheal prevalence.

**Additional file 8.** Results for the association of distance to mine with child health outcomes.

**Additional file 9.** Sensitivity analysis for the interaction effect on water and sanitation infrastructure.

## Data Availability

The datasets supporting the conclusions of this article are available on request from the DHS website (https://dhsprogram.com/).

## Abbreviations

aOR: Adjusted odds ratio
aRRR: Adjusted relative risk ratio
CI: Confidence interval
DHS: Demographic and Health Survey
DiD: Difference-in-difference
HH: Household
GPS: Global Positioning System
OR: Odds ratio
RRR: Relative risk ratio
SDC: Swiss Agency for Development and Cooperation
SDG: Sustainable Development Goal
SNSF: Swiss National Science Foundation
SSA: Sub-Saharan Africa
S&P: Standard & Poor’s
