# Supplementary material for: Impact of mining projects on water and sanitation infrastructures and associated child health outcomes: a multi-country analysis of Demographic and Health Surveys (DHS) in sub-Saharan Africa
Source: Global Health. 2021 Jun 30;17:70. doi: 10.1186/s12992-021-00723-2 (PMC8247184; doi:10.1186/s12992-021-00723-2)
Supplement: Supplementary file 1 — Additional file 1. Equations. [file 12992_2021_723_MOESM1_ESM.docx]

$mlogit(y_{ij}^{\left( m \right)}= \alpha+\beta_{1}^{\left( m \right)}*distance to mine+\beta_{2}^{\left( m \right)}*X_{j}+\varepsilon_{i}^{\left( m \right)})$ (Eq. A1)

$logit(y_{ijk}= \alpha+\beta_{1}*distance to mine+\beta_{2}*X_{j}+\beta_{3}*X_{k}+\varepsilon_{i})$ (Eq. A2)

$mlogit(y_{jl}^{\left( m \right)}= \alpha^{\left( m \right)}+\beta_{1}^{\left( m \right)}*{close}_{j}*{operational}_{jl}+\beta_{2}^{\left( m \right)}*X_{j}+\varepsilon_{l}^{\left( m \right)})$ (Eq. A3)

$logit(y_{ijk}= \alpha+\beta_{1}*{close}_{j}*{operational}_{jl}+\beta_{2}*X_{j}+\beta_{3}*X_{k}+\varepsilon_{l})$ (Eq. A4)
