# Supplementary material for: Impact of mining projects on water and sanitation infrastructures and associated child health outcomes: a multi-country analysis of Demographic and Health Surveys (DHS) in sub-Saharan Africa
Source: Global Health. 2021 Jun 30;17:70. doi: 10.1186/s12992-021-00723-2 (PMC8247184; doi:10.1186/s12992-021-00723-2)
Supplement: Supplementary file 2 — Additional file 2. Percentage of households being classified as wealthy and poor. [file 12992_2021_723_MOESM2_ESM.docx]

**Percentage of households being classified as wealthy and poor.**

|  | **% wealthy households** | |  | **% poorer households** | |
| --- | --- | --- | --- | --- | --- |
|  | **Close (≤10 km)** | **Comparison (10-50 km)** |  | **Close (≤10 km)** | **Comparison (10-50 km)** |
| **Before mine opening** | 35.3 | 36.2 |  | 41.5 | 42.1 |
| **After mine opening** | 70.6 | 29.7 |  | 14.9 | 47.7 |

Wealthy corresponds to the upper two wealth quintiles; the poor belong to the lower two wealth quintiles.
