# Supplementary figures and images for: Impact of mining projects on water and sanitation infrastructures and associated child health outcomes: a multi-country analysis of Demographic and Health Surveys (DHS) in sub-Saharan Africa
Source: Global Health. 2021 Jun 30;17:70. doi: 10.1186/s12992-021-00723-2 (PMC8247184; doi:10.1186/s12992-021-00723-2)

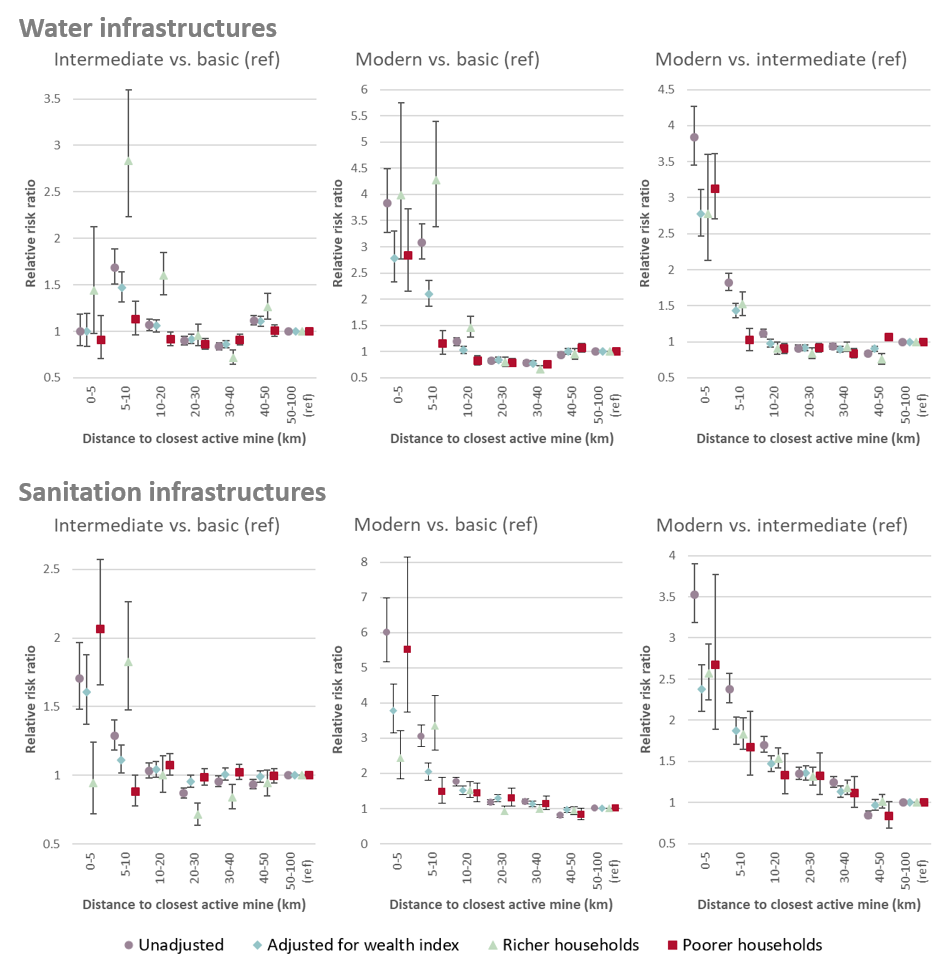

Supplement: Supplementary file 3 — Additional file 3. Spatial trend of the relative risk ratios for the association between the distance to the closest mine and water and sanitation infrastructures. [file 12992_2021_723_MOESM3_ESM.tif]

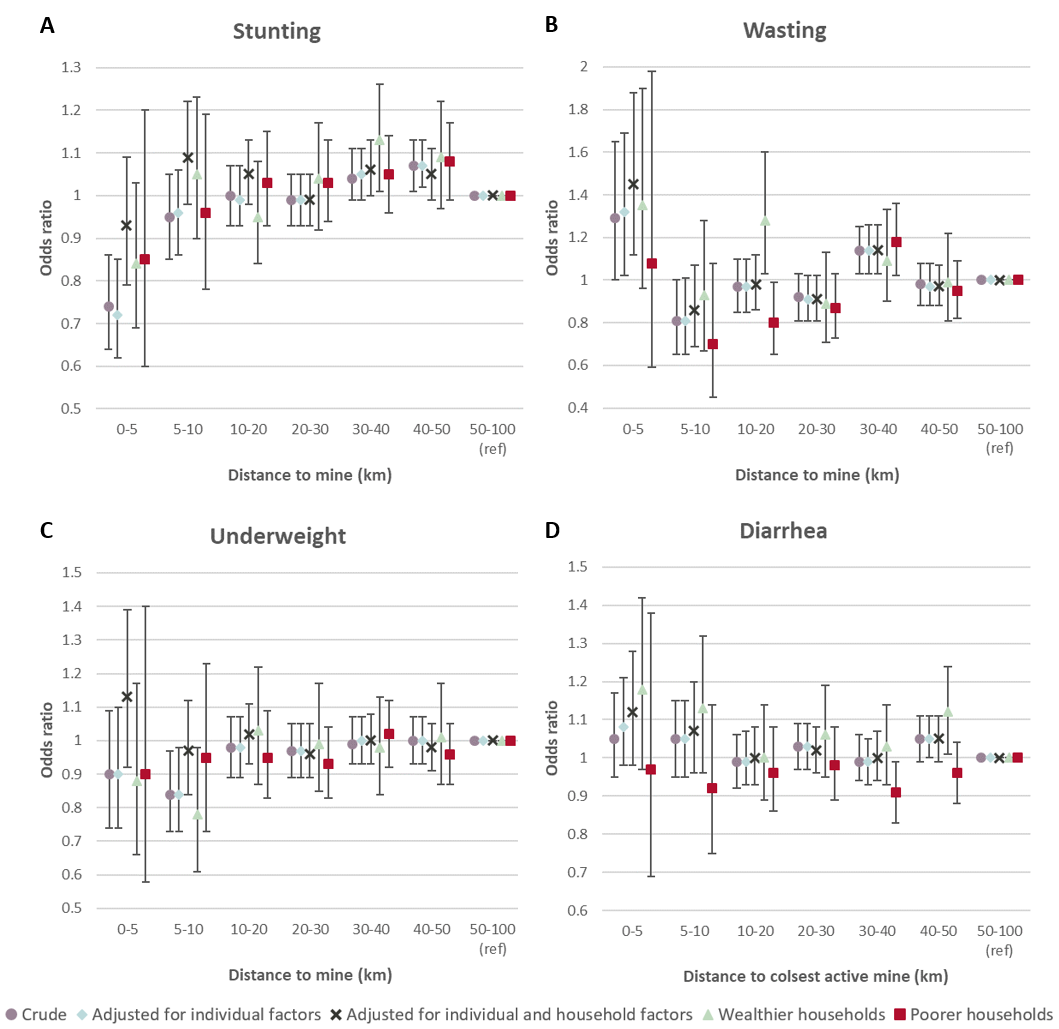

Supplement: Supplementary file 7 — Additional file 7. Spatial trend of the odds ratios for the association between the distance to the closest mine and stunting, wasting, underweight and 2-week diarrheal prevalence. [file 12992_2021_723_MOESM7_ESM.tif]
