# Supplementary material for: Impact of mining projects on water and sanitation infrastructures and associated child health outcomes: a multi-country analysis of Demographic and Health Surveys (DHS) in sub-Saharan Africa
Source: Global Health. 2021 Jun 30;17:70. doi: 10.1186/s12992-021-00723-2 (PMC8247184; doi:10.1186/s12992-021-00723-2)
Supplement: Supplementary file 4 — Additional file 4. Results from the regression models for the association between distance to mine and water infrastructures. [file 12992_2021_723_MOESM4_ESM.docx]

**Results from the regression models for the association between distance to mine and water infrastructures.**

| **Outcome** | ≤5 km (RRR (95% CI)) | 5-10 km (RRR (95% CI)) | 10-20 km  (RRR (95% CI)) | 20-30 km  (RRR (95% CI)) | 30-40 km  (RRR (95% CI)) | 40-50 km  (RRR (95% CI)) | 50-100 km |
| --- | --- | --- | --- | --- | --- | --- | --- |
| Model |  |  |  |  |  |  |  |
| **Intermediate vs.  basic (ref)** |  |  |  |  |  |  |  |
| crude model^†^ | 1.00 (0.42-1.18) | 1.69 (1.51-1.88)** | 1.07 (1.01-1.13)* | 0.90 (0.85-0.95)** | 0.84 (0.80-0.88)** | 1.12 (1.07-1.17)** | 1 (ref) |
| adj. for HH wealth^‡^ | 1.00 (0.84-1.19) | 1.47 (1.31-1.64)** | 1.06 (1.00-1.12) | 0.92 (0.87-0.97)* | 0.86 (0.82-0.90)** | 1.10 (1.05-1.16)** | 1 (ref) |
| wealthier HH only^†^^ | 1.44 (0.98-2.13) | 2.83 (2.23-3.60)** | 1.60 (1.39-1.85)** | 0.95 (0.84-1.08) | 0.72 (0.64-0.80)** | 1.26 (1.13-1.41)** | 1 (ref) |
| poorer HH only^†^^ | 0.91 (0.71-1.17) | 1.13 (0.96-1.32) | 0.92 (0.85-0.99)* | 0.86 (0.81-0.92)** | 0.91 (0.85-0.97)* | 1.01 (0.95-1.07) | 1 (ref) |
| **Modern vs.  basic (ref)** |  |  |  |  |  |  |  |
| crude model^†^ | 3.83 (3.27-4.48)** | 3.08 (2.79-3.44)** | 1.19 (1.12-1.27)** | 0.82 (0.78-0.87)** | 0.79 (0.75-0.82)** | 0.94 (0.89-0.99* | 1 (ref) |
| adj. for HH wealth^‡^ | 2.78 (2.33-3.30)** | 2.10 (1.87-2.35)** | 1.03 (0.97-1.11) | 0.84 (0.79-0.89)** | 0.78 (0.74-0.82)** | 1.00 (0.95-1.05) | 1 (ref) |
| wealthier HH only^†^^ | 3.99 (2.76-5.75)** | 4.27 (3.38-5.39)** | 1.46 (1.27-1.67)** | 0.80 (0.71-0.90)** | 0.66 (0.60-0.73)** | 0.95 (0.85-1.06) | 1 (ref) |
| poorer HH only^†^^ | 2.83 (2.16-3.72)** | 1.16 (0.95-1.40) | 0.83 (0.75-0.92)** | 0.79 (0.72-0.86)** | 0.76 (0.70-0.82)** | 1.07 (0.99-1.16) | 1 (ref) |
| **Modern vs. intermediate (ref)** |  |  |  |  |  |  |  |
| crude model^†^ | 3.84 (3.45-4.27)** | 1.82 (1.71-1.95)** | 1.11 (1.06-1.17)** | 0.91 (0.88-0.96)** | 0.94 (0.90-0.98)* | 0.84 (0.81-0.87)** | 1 (ref) |
| adj. for HH wealth^‡^ | 2.78 (2.47-3.11)** | 1.43 (1.33-1.53)** | 0.98 (0.93-1.03) | 0.92 (0.88-0.96)** | 0.90 (0.86-0.94)** | 0.91 (0.87-0.94)** | 1 (ref) |
| wealthier HH only^†^^ | 2.78 (2.36-3.27)** | 1.52 (1.37-1.68)** | 0.91 (0.84-0.98)* | 0.84 (0.78-0.90)** | 0.93 (0.87-1.00)* | 0.76 (0.71-0.81)** | 1 (ref) |
| poorer HH only^†^^ | 3.12 (2.47-3.94)** | 1.02 (0.87-1.20) | 0.90 (0.82-0.99)* | 0.92 (0.84-0.99)* | 0.84 (0.78-0.90)** | 1.06 (0.99-1.14) | 1 (ref) |

The reported relative risk ratios (RRR) and their corresponding 95% confidence intervals (95% CI) were derived using with multinomial regression models in the generalized structural equation modelling suite in Stata.
† survey-level random intercept only
‡ additionally adjusted for household (HH) wealth quintile
^ stratified analyses using only data from the two lower wealth quintiles (poorer households) and the two upper wealth quintiles (wealthier households), respectively
* *p* < 0.05; ** *p*<0.001
