# Supplementary material for: Impact of mining projects on water and sanitation infrastructures and associated child health outcomes: a multi-country analysis of Demographic and Health Surveys (DHS) in sub-Saharan Africa
Source: Global Health. 2021 Jun 30;17:70. doi: 10.1186/s12992-021-00723-2 (PMC8247184; doi:10.1186/s12992-021-00723-2)
Supplement: Supplementary file 5 — Additional file 5. Results from the regression models for the association between distance to mine and sanitation infrastructures. [file 12992_2021_723_MOESM5_ESM.docx]

**Results from the regression models for the association between distance to mine and sanitation infrastructures.**

| **Outcome** | ≤5 km (RRR (95% CI)) | 5-10 km (RRR (95% CI)) | 10-20 km  (RRR (95% CI)) | 20-30 km  (RRR (95% CI)) | 30-40 km  (RRR (95% CI)) | 40-50 km  (RRR (95% CI)) | 50-100 km |
| --- | --- | --- | --- | --- | --- | --- | --- |
| Model |  |  |  |  |  |  |  |
| **Intermediate vs. basic (ref)** |  |  |  |  |  |  |  |
| crude model^†^ | 1.71 (1.48-1.97)** | 1.29 (1.18-1.40)** | 1.03 (0.98-1.09) | 0.87 (0.83-0.91)** | 0.96 (0.92-0.99)* | 0.94 (0.90-0.97)* | 1 (ref) |
| adj. for HH wealth^‡^ | 1.61 (1.37-1.88)** | 1.11 (1.01-1.22)* | 1.04 (0.99-1.10) | 0.95 (0.91-1.00)* | 1.01 (0.97-1.05) | 0.99 (0.95-1.03) | 1 (ref) |
| wealthier HH only^†^^ | 0.94 (0.72-1.24) | 1.83 (1.47-2.26)** | 1.00 (0.88-1.14) | 0.71 (0.64-0.80)** | 0.84 (0.76-0.93)* | 0.94 (0.85-1.04) | 1 (ref) |
| poorer HH only^†^^ | 2.07 (1.66-2.57)** | 0.88 (0.77-1.00) | 1.08 (1.00-1.16)* | 0.98 (0.93-1.05) | 1.02 (0.97-1.08) | 0.99 (0.94-1.05) | 1 (ref) |
| **Modern vs.  basic (ref)** |  |  |  |  |  |  |  |
| crude model^†^ | 6.00 (5.16-6.98)** | 3.05 (2.76-3.37)** | 1.76 (1.65-1.88)** | 1.18 (1.11-1.25)** | 1.20 (1.13-1.27)** | 0.80 (0.75-0.85)** | 1 (ref) |
| adj. for HH wealth^‡^ | 3.78 (3.15-4.53)** | 2.04 (1.81-2.30)** | 1.52 (1.40-1.65)** | 1.30 (1.20-1.40)** | 1.13 (1.05-1.22)* | 0.96 (0.89-1.03) | 1 (ref) |
| wealthier HH only^†^^ | 2.43 (1.84-3.21)** | 3.35 (2.67-4.21)** | 1.53 (1.33-1.76)** | 0.94 (0.83-1.07) | 0.99 (0.88-1.11) | 0.94 (0.83-1.06) | 1 (ref) |
| poorer HH only^†^^ | 5.52 (3.73-8.15)** | 1.48 (1.15-1.89)* | 1.43 (1.19-1.72)** | 1.30 (1.08-1.58)* | 1.14 (0.96-1.36) | 0.83 (0.69-1.01) | 1 (ref) |
| **Modern vs. intermediate (ref)** |  |  |  |  |  |  |  |
| crude model^†^ | 3.53 (3.19-3.90)** | 2.38 (2.21-2.57)** | 1.70 (1.61-1.80)** | 1.35 (1.28-1.43)** | 1.25 (1.19-1.32)** | 0.85 (0.81-0.90)** | 1 (ref) |
| adj. for HH wealth^‡^ | 2.38 (2.11-2.67)** | 1.87 (1.71-2.04)** | 1.47 (1.38-1.57)** | 1.36 (1.27-1.45)** | 1.13 (1.06-1.20)** | 0.97 (0.91-1.04) | 1 (ref) |
| wealthier HH only^†^^ | 2.57 (2.25-2.93)** | 1.83 (1.65-2.03)** | 1.54 (1.42-1.66)** | 1.31 (1.21-1.43)** | 1.18 (1.10-1.27)** | 1.01 (0.94-1.10) | 1 (ref) |
| poorer HH only^†^^ | 2.67 (1.89-3.77)** | 1.68 (1.33-2.11)** | 1.33 (1.11-1.59)* | 1.32 (1.09-1.6)* | 1.12 (0.94-1.32) | 0.84 (0.69-1.01) | 1 (ref) |

The reported relative risk ratios (RRR) and their corresponding 95% confidence intervals (95% CI) were derived using with multinomial regression models in the generalized structural equation modelling suite in Stata.
† survey-level random intercept only
‡ additionally adjusted for household (HH) wealth quintile
^ stratified analyses using only data from the two lower wealth quintiles (poorer households) and the two upper wealth quintiles (wealthier households), respectively
* *p* < 0.05; ** *p*<0.001
