# Supplementary material for: Impact of mining projects on water and sanitation infrastructures and associated child health outcomes: a multi-country analysis of Demographic and Health Surveys (DHS) in sub-Saharan Africa
Source: Global Health. 2021 Jun 30;17:70. doi: 10.1186/s12992-021-00723-2 (PMC8247184; doi:10.1186/s12992-021-00723-2)
Supplement: Supplementary file 6 — Additional file 6. Sensitivity analysis for the interaction effect on water and sanitation infrastructure. [file 12992_2021_723_MOESM6_ESM.docx]

**Sensitivity analysis for the interaction effect on water and sanitation infrastructure.**

|  | ±2 year time lag^∆^: RRR (95%CI) for interaction close*active | | | |
| --- | --- | --- | --- | --- |
|  | **crude†** (N_water_=27952) (N_sanitation_=28032) | **adjusted‡** (N_water_=27952) (N_sanitation_=28032) | **rich^** (N_water_=10243) (N_sanitation_=10295) | **poor^** (N_water_=11673) (N_sanitation_=11685) |
| Water: modern vs. basic (ref) | 131.43 (82.32 - 209.83)** | 54.93 (33.60 - 89.82)** | 322.89 (71.04 - 1467.54)** | 35.82 (12.56 - 102.13)** |
| Water: intermediate vs. basic (ref) | 14.21 (9.19 - 21.98)** | 8.47 (5.45 - 13.15)** | 27.32 (5.70 - 130.97)** | 4.65 (2.50 - 8.65)** |
| Water: modern vs. intermediate (ref) | 9.21 (5.98 - 14.19)** | 6.49 (4.18 - 10.07)** | 7.89 (4.47 - 13.91)** | 7.70 (2.66 - 22.23)** |
| Sanitation: modern vs. basic (ref) | 17.17 (6.66 - 44.26)** | 4.12 (1.51 - 11.22)* | 25.56 (3.08 - 211.99)* | 0.88 (0.17 - 4.47) |
| Sanitation: interme-diate vs. basic (ref) | 2.03 (1.43 - 2.88)** | 1.24 (0.85 - 1.81) | 1.06 (0.51 - 2.21) | 0.48 (0.26 - 0.88)* |
| Sanitation: modern vs. intermediate (ref) | 8.48 (3.36 - 21.35)** | 3.32 (1.27 - 8.69)* | 24.20 (3.24 - 180.61)* | 1.83 (0.36 - 9.26) |

The coefficients quantify the interaction effect of mining activity (before vs. after mine opening) and proximity to the mine (≤10 km vs. 10-50 km) on access to water and sanitation infrastructures using the longitudinal household dataset. Data during a potential transition phase 2 years before and after mine opening were excluded.
^∆^ sensitivity analysis: data obtained between 2 years before and 2 years after mine opening excluded
† mine-level random intercept only
‡ adjusted for household wealth quintile
^ stratified analyses using only data from the two lower wealth quintiles (poorer households) and the two upper wealth quintiles (wealthier households), respectively
* *p* < 0.05; ** *p*<0.001
