# Supplementary material for: Impact of mining projects on water and sanitation infrastructures and associated child health outcomes: a multi-country analysis of Demographic and Health Surveys (DHS) in sub-Saharan Africa
Source: Global Health. 2021 Jun 30;17:70. doi: 10.1186/s12992-021-00723-2 (PMC8247184; doi:10.1186/s12992-021-00723-2)
Supplement: Supplementary file 8 — Additional file 8. Results for the association of distance to mine with child health outcomes. [file 12992_2021_723_MOESM8_ESM.docx]

**Results for the association of distance to mine with child health outcomes.**

| **Outcome**  Model | **≤5 km (OR (95% CI))** | **5-10 km (OR (95% CI))** | **10-20 km  (OR (95% CI))** | **20-30 km  (OR (95% CI))** | **30-40 km  (OR (95% CI))** | **40-50 km  (OR (95% CI))** | **50-100 km** |
| --- | --- | --- | --- | --- | --- | --- | --- |
| **Stunting** |  |  |  |  |  |  |  |
| crude model^†^ | 0.74 (0.64 - 0.86)** | 0.95 (0.85 - 1.05) | 1.00 (0.93 - 1.07) | 0.99 (0.93 - 1.05) | 1.04 (0.99 - 1.11) | 1.07 (1.01 - 1.13)* | 1 (ref) |
| adj. for ind. factors^‡^ | 0.72 (0.62 - 0.85)** | 0.96 (0.86 - 1.06) | 0.99 (0.93 - 1.07) | 0.99 (0.93 - 1.05) | 1.05 (0.99 - 1.11) | 1.07 (1.02 - 1.13)* | 1 (ref) |
| adj. for ind. and HH factors^∆^ | 0.93 (0.79 - 1.09) | 1.09 (0.98 - 1.22) | 1.05 (0.98 - 1.13) | 0.99 (0.93 - 1.05) | 1.06 (1.00 - 1.13)* | 1.05 (0.99 - 1.11) | 1 (ref) |
| wealthier HH only^‡^^ | 0.84 (0.69 - 1.03) | 1.05 (0.90 - 1.23) | 0.95 (0.84 - 1.08) | 1.04 (0.92 - 1.17) | 1.13 (1.01 - 1.26)* | 1.09 (0.97 - 1.22) | 1 (ref) |
| poorer HH only^‡^^ | 0.85 (0.60 - 1.20) | 0.96 (0.78 - 1.19) | 1.03 (0.93 - 1.15) | 1.03 (0.94 - 1.13) | 1.05 (0.96 - 1.14) | 1.08 (0.99 - 1.17) | 1 (ref) |
| **Wasting** |  |  |  |  |  |  |  |
| crude model^†^ | 1.29 (1.00 - 1.65)* | 0.81 (0.65 - 1.00)* | 0.97 (0.85 - 1.10) | 0.92 (0.81 - 1.03) | 1.14 (1.03 - 1.25)* | 0.98 (0.88 - 1.08) | 1 (ref) |
| adj. for ind. factors^‡^ | 1.32 (1.02 - 1.69)* | 0.81 (0.65 - 1.01) | 0.97 (0.85 - 1.10) | 0.91 (0.81 - 1.02) | 1.14 (1.03 - 1.26)* | 0.97 (0.88 - 1.08) | 1 (ref) |
| adj. for ind. and HH factors^∆^ | 1.45 (1.12 - 1.88)* | 0.86 (0.69 - 1.07) | 0.98 (0.86 - 1.12) | 0.91 (0.81 - 1.02) | 1.14 (1.03 - 1.26)* | 0.97 (0.88 - 1.07) | 1 (ref) |
| wealthier HH only^‡^^ | 1.35 (0.96 - 1.90) | 0.93 (0.67 - 1.28) | 1.28 (1.03 - 1.60)* | 0.89 (0.71 - 1.13) | 1.09 (0.90 - 1.33) | 0.99 (0.81 - 1.22) | 1 (ref) |
| poorer HH only^‡^^ | 1.08 (0.59 - 1.98) | 0.70 (0.45 - 1.08) | 0.80 (0.65 - 0.99)* | 0.87 (0.73 - 1.03) | 1.18 (1.02 - 1.36)* | 0.95 (0.82 - 1.09) | 1 (ref) |
| **Underweight** |  |  |  |  |  |  |  |
| crude model^†^ | 0.90 (0.74 - 1.09) | 0.84 (0.73 - 0.97) | 0.98 (0.89 - 1.07) | 0.97 (0.89 - 1.05) | 0.99 (0.93 - 1.07) | 1.00 (0.93 - 1.07) | 1 (ref) |
| adj. for ind. factors^‡^ | 0.90 (0.74 - 1.10) | 0.84 (0.73 - 0.98) | 0.98 (0.89 - 1.07) | 0.97 (0.89 - 1.05) | 1.00 (0.93 - 1.07) | 1.00 (0.93 - 1.07) | 1 (ref) |
| adj. for ind. and HH factors^∆^ | 1.13 (0.92 - 1.39) | 0.97 (0.84 - 1.12) | 1.02 (0.93 - 1.11) | 0.96 (0.89 - 1.05) | 1.00 (0.93 - 1.08) | 0.98 (0.91 - 1.05) | 1 (ref) |
| wealthier HH only^‡^^ | 0.88 (0.66 - 1.17) | 0.78 (0.61 - 0.98)* | 1.03 (0.87 - 1.22) | 0.99 (0.85 - 1.17) | 0.98 (0.84 - 1.13) | 1.01 (0.87 - 1.17) | 1 (ref) |
| poorer HH only^‡^^ | 0.90 (0.58 - 1.40) | 0.95 (0.73 - 1.23) | 0.95 (0.83 - 1.09) | 0.93 (0.83 - 1.04) | 1.02 (0.92 - 1.12) | 0.96 (0.87 - 1.05) | 1 (ref) |
| **Diarrhea** |  |  |  |  |  |  |  |
| crude model^†^ | 1.05 (0.91 - 1.21) | 1.05 (0.94 - 1.16) | 0.99 (0.92 - 1.07) | 1.03 (0.96 - 1.09) | 0.99 (0.93 - 1.05) | 1.05 (0.99 - 1.11) | 1 (ref) |
| adj. for ind. factors^‡^ | 1.08 (0.93 - 1.25) | 1.05 (0.94 - 1.17) | 0.99 (0.92 - 1.06) | 1.03 (0.96 - 1.09) | 0.99 (0.93 - 1.05) | 1.05 (0.99 - 1.11) | 1 (ref) |
| adj. for ind. and HH factors^∆^ | 1.12 (0.96 - 1.31) | 1.07 (0.96 - 1.20) | 1.00 (0.93 - 1.07) | 1.02 (0.95 - 1.08) | 1.00 (0.94 - 1.06) | 1.05 (0.99 - 1.11) | 1 (ref) |
| wealthier HH only^‡^^ | 1.18 (0.97 - 1.42) | 1.13 (0.96 - 1.32) | 1.00 (0.89 - 1.14) | 1.06 (0.95 - 1.19) | 1.03 (0.93 - 1.14) | 1.12 (1.01 - 1.24)* | 1 (ref) |
| poorer HH only^‡^^ | 0.97 (0.69 - 1.38) | 0.92 (0.75 - 1.14) | 0.96 (0.86 - 1.08) | 0.98 (0.89 - 1.08) | 0.91 (0.83 - 0.99)* | 0.96 (0.88 - 1.04) | 1 (ref) |

Households at a distance between 50 and 100 km were used as reference category.
† survey-level random intercept only
‡ adjusted for individual-level factors (child age and sex)
^ adjusted for individual and household-level factors (wealth, access to water and sanitation, household size)
* *p* < 0.05; ** *p*<0.001
