# Supplementary material for: Impact of mining projects on water and sanitation infrastructures and associated child health outcomes: a multi-country analysis of Demographic and Health Surveys (DHS) in sub-Saharan Africa
Source: Global Health. 2021 Jun 30;17:70. doi: 10.1186/s12992-021-00723-2 (PMC8247184; doi:10.1186/s12992-021-00723-2)
Supplement: Supplementary file 9 — Additional file 9. Sensitivity analysis for the interaction effect on water and sanitation infrastructure. [file 12992_2021_723_MOESM9_ESM.docx]

**Sensitivity analysis for the interaction effect on water and sanitation infrastructure.**

|  | ±2 year time lag^∆^: OR (95%CI) for interaction close*active | | | | |
| --- | --- | --- | --- | --- | --- |
|  | crude model^†^ | adj. for ind. factors^‡^ | adj. for ind. and HH factors^∞^ | wealthier HH only^‡^^ | poorer HH only^‡^^ |
| Stunting | 0.63  (0.41 - 0.98)* | 0.63  (0.40 - 0.99)* | 0.85  (0.54 - 1.34) | 0.65  (0.28 - 1.53) | 0.73  (0.34 - 1.54) |
| Wasting | 0.51  (0.27 - 0.98)* | 0.48  (0.25 - 0.94)* | 0.55  (0.28 - 1.07) | 1.15  (0.23 - 5.88) | 0.32  (0.12 - 0.82)* |
| Underweight | 0.55 (0.34 - 0.90)* | 0.56 (0.34 - 0.91)* | 0.79 (0.48 - 1.30) | 1.49 (0.46 - 4.81) | 0.43 (0.20 - 0.93)* |
| Diarrhea | 0.82  (0.56 - 1.20) | 0.81  (0.54 - 1.19) | 0.85  (0.57 - 1.27) | 1.33  (0.63 - 2.81) | 0.42  (0.21 - 0.83)* |

The coefficients quantify the interaction effect of mining activity (before vs. after mine opening) and proximity to the mine (≤10 km vs. 10-50 km) on childhood health outcomes using the longitudinal household dataset. Data during a potential transition phase 2 years before and after mine opening were excluded.
^∆^ sensitivity analysis: data obtained between 2 years before and 2 years after mine opening excluded
† mine-level random intercept only
‡ adjusted for individual-level factors (child age and sex)
**^∞^** adjusted for individual and household-level factors (wealth, access to water and sanitation, household size)
^ stratified analyses using only data from the two lower wealth quintiles (poorer households) and the two upper wealth quintiles (wealthier households), respectively
* *p* < 0.05; ** *p*<0.001
